# Influence of Flow Rate, Particle Size, and Temperature on Espresso Extraction Kinetics

**DOI:** 10.3390/foods12152871

**Published:** 2023-07-28

**Authors:** Benedikt K. L. Schmieder, Verena B. Pannusch, Lara Vannieuwenhuyse, Heiko Briesen, Mirjana Minceva

**Affiliations:** 1 Biothermodynamics, TUM School of Life Sciences, Technical University of Munich, 85354 Freising, Germany; 2 Process Systems Engineering, TUM School of Life Sciences, Technical University of Munich, 85354 Freising, Germany

**Keywords:** espresso, coffee, extraction kinetics, non-volatiles, caffeine, trigonelline, caffeoylquinic acid, brew ratio

## Abstract

Brewing espresso coffee (EC) is considered a craft and, by some, even an art. Therefore, in this study, we systematically investigated the influence of coffee grinding, water flow rate, and temperature on the extraction kinetics of representative EC components, employing a central composite experimental design. The extraction kinetics of trigonelline, caffeine, 5-caffeoylquinic acid (5-CQA), and Total Dissolved Solids (TDS) were determined by collecting and analyzing ten consecutive fractions during the EC brewing process. From the extraction kinetics, the component masses in the cup were calculated for Ristretto, Espresso, and Espresso Lungo. The analysis of the studied parameters revealed that flow rate had the strongest effect on the component mass in the cup. The intensity of the flow rate influence was more pronounced at finer grindings and higher water temperatures. Overall, the observed influences were minor compared to changes resulting from differences in total extracted EC mass.

## 1. Introduction

Espresso coffee (EC) brewing is a solid–liquid extraction process from a packed (coffee) bed—the coffee puck [1]. A commonly recommended extraction method for the preparation of EC utilizes a pump (9 ± 2 bar) to infuse 6 ± 1.5 g fine ground and tamped coffee in a portafilter with heated water (90 ± 5 °C). The typical extraction for a single espresso (25 ± 5 mL) takes approximately 30 ± 5 s. The ranges of these recommendations for EC extraction leave much leeway to the barista, as the complex influence of the operating parameters on the final product is not fully understood. Hence, brewing espresso coffee is a craft, even considered an art by some.

EC is obtained from only two main ingredients: water and ground, roasted coffee beans. The type and quality of the used coffee beans and water set the quality window attainable by the EC extraction. The most common coffee bean species *Coffea arabica* (Arabica) and *Coffea canephora* (Robusta) differ significantly in their chemical, physical, and sensory properties [2]. Additionally, the post-harvest processes [3], storage [4] and the degree of roasting [5,6] change the chemical compositions of the beans through thermal degradation or neogenesis of new compounds, e.g., by the Maillard reaction [7]. The water mineral content and composition also influence the EC extraction [8,9].

Most scientific studies on EC extraction focus on analyzing sensory attributes, volatile and non-volatile EC components. Over 1000 components are present in an EC cup, of which around 30–50 are considered key odorants [10,11]. For non-volatile components, typically, trigonelline, caffeine, caffeoylquinic acids, caffeoylquinic acid lactones, organic acids, fatty acids, and lipids, are analyzed [12]. The main aspects considered for an EC extraction are grinding and tamping the coffee in the portafilter, the water flow rate, pressure, and temperature, as well as the extraction time that determines the EC mass in the cup [12]. The ratio between the ground coffee mass in the puck and the extracted EC mass in the cup is called the brew ratio (BR).

The grinding process defines the particle size distribution and the amount of coffee mass in the portafilter. Finer grinding levels (GL) increase the particle surface area in contact with water, enabling a higher extraction yield for trigonelline, caffeine, and 5-caffeoylquinic acid (5-CQA) [13,14,15,16,17]. Increasing the ground coffee mass at a similar extracted EC volume increases the masses of trigonelline, caffeine, and 5-CQA in the cup [18,19]. Particle size distribution, coffee mass, and tamping define the puck’s mechanical structure and hydraulic resistance, which, in turn, sets the relation of water flow rate and pressure [20]. The relation between water pressure and flow rate can be described by Darcy’s law [21].

In previous studies, increasing the applied pressure from 7 to 11 bar has shown a decreasing trend in extracted component mass in the coffee cup [22,23,24]. The corresponding flow rates (F) for the pressure-controlled experiments were not reported. Though, Lee et al. pointed out that the flow through a coffee puck is non-uniform and could lead to irregular EC extractions [25].

Higher water temperatures (T) increase the components’ solubility and reduce water viscosity [26]. However, the influence of water temperature on EC component mass in the cup has been inconclusive for experiments with otherwise constant conditions [13]. Albanese et al. [27] analyzed coffee pods and reported increased caffeine concentration with rising temperatures from 90 °C to 110 °C. Masella et al. [28] found no significant difference in trigonelline, caffeine, and chlorogenic acid concentrations in the EC cup for 75 °C, 80 °C, or 85 °C. For similar EC components, Andueza et al. [29] reported several ambiguous temperature correlations for significant differences between EC brewed at 88 °C, 92 °C, 96 °C, and 98 °C. Also, Salamanca et al. [30] described different influences on the caffeine and 5-CQA concentrations in the cup for upward and downward temperature gradients between 88 °C and 93 °C without identifying a conclusive correlation.

While most studies analyze full EC cups, some authors have highlighted the importance of understanding extraction kinetics, as the concentration of extracted components strongly changes over time. Generally, the majority of EC components are extracted at the beginning of the brew [24,31,32]. Only a few studies compared extraction kinetics for varying preparation and processing conditions. Kuhn et al. [14] and Severini et al. [33] compared the extraction kinetics for different particle size distributions. They observed that trigonelline and caffeine were extracted faster from smaller rather than larger particles.

Overall, there is a highly complex interplay of several preparation and processing parameters on the EC component masses in the cup. Though known to be decisive, only very few data on extraction kinetics for controlled varying conditions are available. This study combines a rigorous statistical experimental design (central composite) with a detailed analysis of time-dependent extraction behaviors (extraction kinetics). This work aims to investigate the individual and combined influences of the extraction process parameters flow rate (F), coffee grinding level (GL), and water temperature (T) on trigonelline, caffeine, 5-caffeoylquinic acid (5-CQA), and Total Dissolved Solids (TDS) masses in the EC cup for Ristretto (BR 1/1), Espresso (BR 1/2), and Espresso Lungo (BR 1/3).

## 2. Materials and Methods

### 2.1. Chemicals

Trigonelline hydrochloride (≥97.5% purity), caffeine (≥99.0%), and 5-caffeoylquinic acid (≥96%) analytical standards were purchased from Sigma-Aldrich Chemie GmbH (Taufkirchen, Germany). HPLC-water (≥99.9%, HiPerSolv Chromanorm), methanol (≥99.9%, HiPerSolv Chromanorm Reag. Ph. Eur.), and formic acid (≥98%, AnalaR Normapur) for the high-performance liquid chromatography (HPLC) analysis were acquired from VWR Chemicals GmbH (Darmstadt, Germany).

### 2.2. Coffee Beans and Roasting

The coffee beans were obtained from List + Beisler GmbH (Hamburg, Germany). The brand *Colombia Suprema Huila* consisted of 100% Arabica and was washed in a post-harvest process. For the experiment, 5 kg was roasted by BB Coffee Company GmbH (Unterhaching, Germany). The coffee beans were roasted in a CRS-30 roaster (Joper SA, Canelas, Portugal) with an increasing temperature profile from 180 °C to 212 °C (190 °C at first crack) for approximately 10 min. Two roasting batches of the same coffee beans were used for the experiments. Both roasting batches were tested in triplicate under the same extraction conditions (20 g puck, flow rate F 2.0 mL s^−1^, grinding level GL 1.7, temperature T 89 °C, 40 g EC) and showed no significant differences for trigonelline, caffeine, 5-CQA, and TDS concentrations in the espresso coffee (EC) cup. After roasting, the coffee beans were stored in 250 g packages for two weeks. The evening before each experiment, the required 250 g packages were opened and divided into air–sealed 50 g packs per EC extraction to prevent aroma loss over the course of the day.

### 2.3. Brewing Water

Bottled 750 mL water *Acqua Panna* (Sanpellegrino S.p.A., Pellegrino Terme, Italy) with the following composition was used to prepare the coffee samples: 106 mg L^−1^ ${HCO}_{3}^{-}$, 32.2 mg L^−1^ ${Ca}_{}^{2+}$, 22.0 mg L^−1^ ${SO}_{4}^{2-}$, 7.8 mg L^−1^ ${Cl}^{-}$, 6.9 mg L^−1^ ${SiO}_{2}$, 6.6 mg L^−1^ ${{Na}^{+}}_{}^{}$, and 6.5 mg L^−1^ ${Mg}^{2+}$.

### 2.4. Coffee Puck Preparation

**Figure A1:**
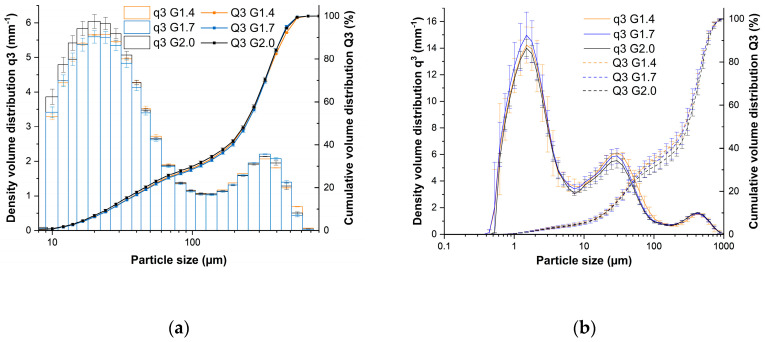
Density volume distribution q3 and cumulative volume distributions Q3 for Mahlkoenig EK65S grinding levels GL 1.4, GL 1.7, and GL 2.0, measured (**a**) dry and (**b**) wet.

The coffee beans were ground on the Mahlkönig E65S (Hemro International AG, Zurich, Switzerland). As EC is typically brewed using fine ground coffee, the grinder was set to the EC grinding levels GL 1.4, GL 1.7, and GL 2.0. The chosen interval corresponded to 7.5% of the available scale, for which the manufacturer declared a volume mean diameter bandwidth of 180–580 µm [34]. The particle size distributions for the used grinding levels were measured dry and wet by laser diffractometer (Helos/BR + Rodos/Quixel, Sympatec GmbH, Clausthal-Zellerfeld, Germany) and showed high similarity (Appendix A Figure A1). The wet-measured, volume-based De Broucker mean particle diameter (standard deviation SD) for the GL 1.4, GL 1.7, and GL 2.0 increased from 273 µm (SD 7.6) to 277 µm (SD 17.0) and 295 µm (SD 18). The respective surface-area-based Sauter mean diameters were 28.3 µm (SD 1.6), 26.9 µm (SD 3.2), and 29.2 µm (SD 1.4) for GL 1.4, GL 1.7, and GL 2.0.

The mass of ground coffee for all experiments was 20 ± 0.01 g. The ground coffee was distributed and levelled with the distribution tool Grande TRE (Sahdia Enterprises GmbH, Frankfurt, Germany). The levelled puck was then tamped parallel to the basket bottom with a force equal to 25 kg on the tamping station CPS Tamper (Macap SRL, Maerne, Italy).

### 2.5. Espresso Coffee Preparation

The extraction was performed with a Decent DE1 Pro (Decent Espresso Intl. Ltd., Hong Kong, China). The machine was equipped with an IMS Cl 200 IM shower screen attached to the original Decent shower head and an IMS BT702Th26.5M precision portafilter basket (I.M.S. spa, Torre D’isola, Italy). During the coffee extraction, ten fractions were collected with a time-controlled sampling wheel developed by Kuhn et al. [14]. The velocity of the sampling wheel was chosen so that ten fractions of similar mass were collected for an average total EC mass of 58.1 g (SD 4.1) for each of the studied flow rates. The average mass of a single fraction was 6.0 g (SD 0.9). Fraction 1 exhibited the highest mass variation because of inaccuracies in starting the sample wheel on the first EC drop. The sampling wheel was positioned on a KB 2400-2N digital scale (Kern & Sohn GmbH, Balingen, Germany) to record the extracted EC mass continuously.

**Figure A2:**
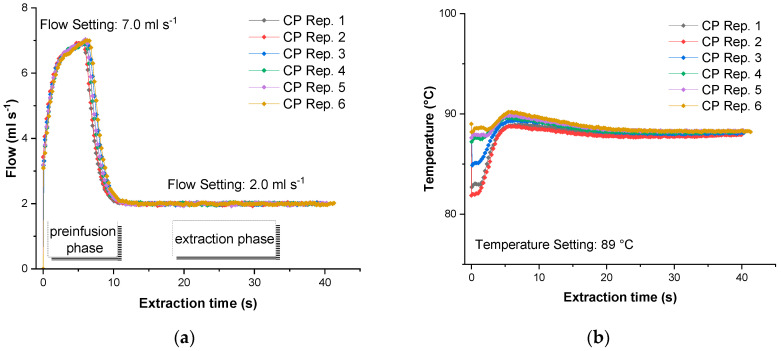
Representative (**a**) flow and (**b**) temperature courses during the espresso coffee preparation with the Decent DE1 Pro for a set preinfusion flow rate of 7.0 mL s^−1^ and for the extraction flow rate of 2.0 mL s^−1^ as well as a temperature of 89 °C.

Water temperature, flow rate, or pressure profiles could be set on the Decent DE1 Pro. The machine measured the brew temperature just above the coffee puck and the brew pressure between the boiler and portafilter. In the experiments performed in this work, the extraction was flow rate controlled in order to keep a constant residence time and to be able to collect fractions of constant mass. In the preinfusion phase, the coffee machine was set to 7 mL s^−1^ and to a preselected temperature (80 °C, 89 °C, or 98 °C), which matched the one used in the extraction phase. The machine changed to the extraction phase setting when the portafilter basket was filled, and the pressure rose above 2.5 bar. For the set water flow rates 1.0 mL s^−1^, 2.0 mL s^−1^, and 3.0 mL s^−1^, the achieved average flow rates of 0.96 mL s^−1^ (SD 0.1), 1.9 mL s^−1^ (SD 0.0), and 2.8 mL s^−1^ (SD 0.1) were determined based on the scale’s time-stamped measurements of the extracted EC mass. The set water temperatures of 80 °C, 89 °C, and 98 °C resulted in average brew temperatures of 79.1 °C (SD 0.5), 88.2 °C (SD 0.5), and 96.5 °C (SD 0.5), as continuously measured by the Decent DE1 Pro. An example of water flow and temperature course during the EC preparation is shown in Appendix A Figure A2. Before each EC extraction experiment, one test EC was brewed with identical settings to pre-heat the machine.

Fractions 1, 2, 3, 5, 7, and 10 were cooled immediately after the extraction in an ice bath. For HPLC analysis, fractions 1 and 2 were diluted with HPLC-water by mass ratios of 1:50, fractions 3 and 5 were diluted by 1:20, and fractions 7 and 10 were diluted by 1:5. The diluted samples were filtered with 0.2 µm Chromafil PET-20/15 MS syringe filters (Macherey-Nagel GmbH & Co., KG, Düren, Germany), and an aliquot of 1.5 mL was stored in a refrigerator at 9 °C until the analysis. For the TDS analysis, 2 mL per fraction was centrifuged at 4700 rpm for 10 min in the Centrifuge 5804 R (Eppendorf AG, Hamburg, Germany), and 0.1 mL of the supernatant was stored in a freezer at −20 °C.

### 2.6. HPLC Analysis

Trigonelline, caffeine, and 5-CQA were analyzed by high performance liquid chromatography (HPLC) on an Agilent 1290 Infinity LC System (Agilent Technologies Inc., Santa Clara, CA, USA) equipped with a UV/VIS detector. The analysis was performed with the reverse phase column VDSpher PUR C18-E (150 mm × 4.6 mm, 5 µm; VDS optilab Chromatographie Technik GmbH, Berlin, Germany). The method described by Farah et al. [5] was modified for the analysis. Eluent A consisted of HPLC-water with 0.5% formic acid and eluent B of methanol with 0.5% formic acid. At the constant flow rate of 1.2 mL min^−1^, the following gradient method was used for 10 µL injected sample volume: 2% B (0–1.2 min), 20% B (2.5 min), 40% B (13 min), 95% B (14.5–15 min), 2% B (15.5–21 min). Trigonelline and caffeine were detected at λ = 272 nm, whereas 5-CQA was detected at λ = 324 nm. The component concentrations were calculated by preparing calibration curves from two stock solutions of the corresponding standards with five calibration points each.

### 2.7. Determination of TDS

The centrifuged (4700 rpm, 10 min) and frozen samples of 0.1 mL were thawed at room temperature and diluted by volume ratio 1:3 with demineralized water from a Milli-Q Direct 8 (Merck KGaA, Darmstadt, Germany). To determine the Total Dissolved Solids (TDS) mass, the limit angle and refractive index at λ = 589 nm and 20 °C were measured in the refractometer DR6000-T (A. Kruess Optronic GmbH, Hamburg, Germany). The calibration was performed according to the German norm DIN 10775 [35] by correlating the refractive index with the mass of dried samples.

### 2.8. Data Processing and Statistical Analysis

#### 2.8.1. Extraction Kinetics Fitting

To characterize the extraction kinetics, the extract’s component concentration at the portafilter outlet was considered as a function of the cumulative extracted EC mass. As no continuous measure of the concentration at the outlet was available, such a function was derived from six analyzed EC fractions (1, 2, 3, 5, 7, and 10). For the discrete samples, the accumulated extracted EC mass until fraction *n* was calculated according to:

(1) $$m_{\sum }^{n}=0.5*m_{n}+\sum_{1}^{n-1}m_{n}$$

The extraction kinetic $c(m_{\sum })$ for continuous accumulated EC mass $m_{\sum }$ was obtained by least-square fitting the discrete concentrations of the analyzed components using the following exponential function:

(2) $$c(m_{\sum })=c_{0}\cdot e^{-\frac{m_{\sum }}{\lambda }}$$

The accumulated extraction mass $m_{\sum }$ was utilized to describe the EC extraction progress and highly correlated with the extraction time for flow-controlled EC brewing. Therefore, *λ* could be interpreted as a time constant. The theoretical start concentration was represented by $c_{0}$. Extraction kinetics were determined individually for every single experimental run. Additionally, for each of the 15 experiment settings presented in Section 2.8.3., the average extraction kinetics were determined by fitting Equation (2) to data obtained in triplicate (6 replicates at DoE central point).

#### 2.8.2. Calculation of Component Mass in EC Cup

From the extraction kinetics, it is possible to calculate the component mass in the EC cup for different beverage sizes at their respective brew ratios (BR). In this study, the influence of flow rate, coffee grinding level, and water temperature on the component mass in the cup for Ristretto (~BR 1/1), Espresso (~BR 1/2), and Espresso Lungo (~BR 1/3) are discussed. Thus, beverage masses of 20 g, 40 g, and 60 g were chosen for the calculation to match the coffee puck mass of 20 ± 0.01 g and achieve the brew ratios of BR 1/1, BR 1/2, and BR 1/3.

The exact extraction kinetic at the beginning of the brew ($m_{cup}\ll m_{Frak.1}$) was unknown and suspected to deviate from the exponential decay described by Equation (2) [36]. Therefore, to calculate the component mass in the cup, the discrete component mass in the first fraction was combined with the mass obtained by integration of the extraction kinetics curve:

(3) $$m_{cup}^{BR}=m_{Frak.1}\;\cdot \;c_{Frak.1}+\int_{m_{Frak.1}}^{20\;g/BR}c\left(m_{\sum }\right)\;dm_{\sum }\;\mathrm{with}\;BR\;∊\;\{1/1,\;1/2,\;1/3\}$$

The component mass of the first fraction was determined by multiplying the measured fraction mass $m_{Frak.1}$ and component concentration in this fraction $c_{Frak.1}$. For the remaining duration of the extraction process, the component mass was determined by integrating the extraction kinetic curve $c(m_{\sum })$, correlated with Equation (2), from the first fraction mass $m_{Frak.1}$ to the desired end mass of the EC beverage.

#### 2.8.3. Statistical Analysis

The influence of flow rate, grinding level, and temperature on the extraction kinetics of trigonelline, caffeine, 5-CQA, and TDS was studied. The experiment set was defined using a face-centered Central Composite Design [37]. Table 1 provides the design of experiment (DoE) operating conditions for the 15 experiments (each with 3 repetitions, 6 repetitions for the central point).

The masses of trigonelline, caffeine, 5-CQA, and TDS in the EC cup ($m_{cup}$) for three different brew ratios (BR 1/1, BR 1/2, and BR 1/3) were used as response variables to evaluate the influence of the studied process parameters flow rate, grinding level, and temperature on the EC extraction. The necessary 12 response sets were calculated with Equation (3) and evaluated by response surface methodology in OriginPro 2021b (OriginLab Corporation, Northampton, MA, USA). The response surface method was based on the following full-quadratic model function:

(4) $$m_{cup}=\beta_{0}+\beta_{1}x_{flow}+\beta_{2}x_{grind}+\beta_{3}x_{temp}+\beta_{4}x_{flow}^{2}+\beta_{5}x_{grind}^{2}+\beta_{6}x_{temp}^{2}\;+\beta_{7}x_{flow}x_{grind}+\beta_{8}x_{flow}x_{temp}+\beta_{9}x_{grind}x_{temp}$$

For the evaluation, the set grinding levels $x_{grind}$ as well as the experimental values for the flow rate $x_{flow}$ and the temperature $x_{temp}$ were used. The coefficient $\beta_{0}$ is the intercept, $\beta_{1}$–$\beta_{3}$ are the linear coefficients, $\beta_{4}$–$\beta_{6}$ are the quadratic coefficients, and $\beta_{7}$–$\beta_{9}$ are the interactive coefficients. The significance of each effect was determined by ANOVA. Based on the ANOVA and the standardized effects, backward elimination was used to reduce the full-quadratic fitting to the significant effect parameters for higher-order factors (significance level α = 0.05) [38].

## 3. Results & Discussion

### 3.1. Extraction Kinetics

**Figure 1:**
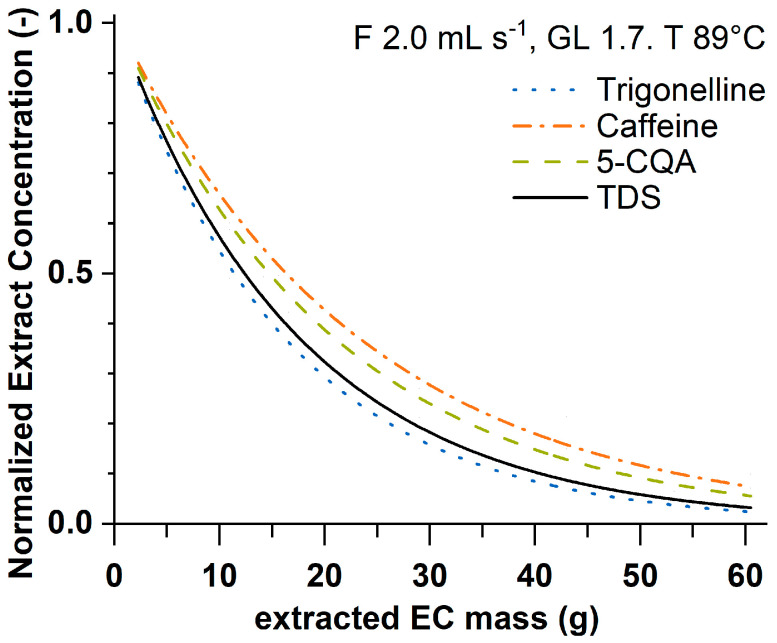
Normalized (with respect to corresponding initial concentration $c_{0}$ from Equation (2)) extraction kinetics for trigonelline, caffeine, 5-CQA, and TDS for the DoE central point (F 2.0 mL s^−1^, GL 1.7, and T 89 °C).

The extraction kinetics describe the components’ concentration change in the espresso coffee (EC) extract as a function of the cumulative extracted EC mass in the cup. The extract concentration of all compounds was highest at the beginning of the brewing process (i.e., in the first collected fraction) and decreased exponentially the more EC was extracted. The fastest decrease was observed for trigonelline and Total Dissolved Solids (TDS), followed by 5-caffeoylquinic acid (5-CQA) and caffeine. This behavior was also described in the literature and correlated with the components’ polarity for trigonelline, caffeine, and 5-CQA [39,40]. For comparison, Figure 1 shows the extraction kinetic curves normalized with respect to the corresponding initial concentration $c_{0}$ for different components obtained by fitting Equation (2) to the experimental data of the design of experiment’s (DoE) central point.

**Figure 2:**
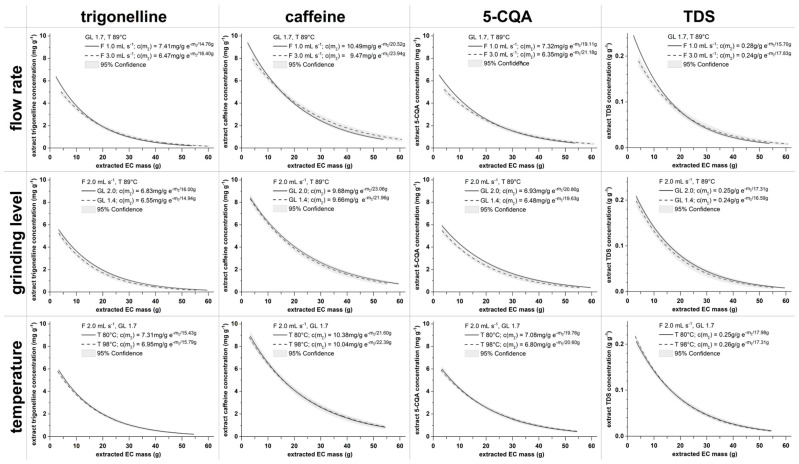
Trigonelline, caffeine, 5-CQA, and TDS extraction kinetics $c(m_{\sum })$ for lower and upper DoE boundary settings of water flow rate (F), grinding level (GL), and temperature (T), whereas the remaining process parameters were kept at DoE central point settings (F 2.0 mL s^−1^, GL 1.7, and T 89 °C).

For all 15 performed experiments, the fit parameters of the average extraction kinetic curves can be found in Appendix A Table A1. In addition, the raw concentration data for the fractions 1, 2, 3, 5, 7, and 10 for all experiments’ replicates are available in Supplementary Materials Table S1. For the experiments at the DoE axis points (Exp. 1–6 in Table 1), the average extraction kinetics for the concentration of trigonelline, caffeine, 5-CQA, and TDS in the EC extract are shown in Figure 2. At the DoE axis points, only one process parameter is changed at a time compared to the DoE central point settings (F 2.0 mL s^−1^, GL 1.7, and T 89 °C). The process parameters are set to the lower and upper boundaries of the DoE space (see Table 1). In general, the studied components show similar behaviors toward the different influences of the process parameters flow rate (F), grinding level (GL), and temperature (T).

The component extraction kinetics for the flow rates at the lower and upper boundaries (Figure 2, top row) differ at the beginning of the extraction and in their extraction dynamics. The extract’s component concentrations at the beginning are higher for the slower flow rate of 1.0 mL s^−1^ but decrease faster during the brew than for the faster flow rate of 3.0 mL s^−1^. The difference in the extract’s component concentrations between the slow and fast flow rates decreases as the extraction progresses. A slower flow rate allows for a longer contact time between water and ground coffee. As the extraction process is time dependent, the longer contact time explains the higher concentrations of the EC extract at the beginning of the brew [1].

The extraction kinetics obtained for the lower and upper boundaries of the grinding level (Figure 2, middle row) behave similarly considering their extraction rates. However, the lower grinding level GL 1.4 extract’s component concentration is slightly smaller than GL 2.0. Their 95% confidence bands overlap for most of the brewing process.

The obtained extraction kinetics are nearly identical for the temperature lower and upper boundary settings (Figure 2, bottom row), and their 95% confidence bands overlap for the whole analyzed brewing process. Consequently, in contrast to the literature [23,30], no measurable influence on the trigonelline, caffeine, 5-CQA, and TDS masses in the cup would be expected by individually changing the water temperatures from 80 °C to 98 °C.

### 3.2. Extracted Component Mass in the Cup for Brew Ratios 1/1, 1/2, and 1/3

The average trigonelline, caffeine, 5-CQA, and TDS masses in the EC cup for brew ratios BR 1/1, BR1/2, and BR1/3 are presented in Table 2, together with the set grinding levels and the measured process parameters flow rates, temperatures, and pressures. The presented component masses in the cup are average values for each of the 15 experiments based on integrating each replicate’s extraction kinetics individually with Equation (3) for extracted EC mass of 20 g (BR 1/1), 40 g (BR 1/2), and 60 g (BR 1/3). The parameters for the extraction kinetic curves for the individual replicates can be found in Supplementary Materials Table S3.

The overall average concentrations for all experiments for a 40 g EC cup (BR 1/2) were 2.45 mg g^−1^ for trigonelline, 4.57 mg g^−1^ for caffeine, 2.96 mg g^−1^ for 5-CQA, and 9.68 g (100 g)^−1^ for TDS. The use of different coffee and water types, roasting levels, coffee puck masses, beverage sizes, and extraction machines impeded quantitative comparisons to other studies. Angeloni et al., who also used a 20 g Arabica coffee puck and a ~BR 1/2, extracted, on average, 3.39 mg g^−1^ trigonelline, 5.18 mg g^−1^ caffeine, 5.27 mg g^−1^ 5-CQA, and 10.02 g (100 g)^−1^ TDS [13]. Taking into consideration that Caprioli et al. reported trigonelline masses in the cup ranging from 28.20 mg to 65.08 mg and caffeine masses from 116.87 mg to 199.68 mg for the same extraction settings (7.5 g ground coffee, 25 mL EC, 25 s) for 20 different EC coffee brands, the experimental results were within the expected range [24]. Additionally, 5-CQA is known to be affected strongly by different roasting processes [41,42], which could further explain the concentration differences between the two studies. For TDS, the measured values were in accordance with Angeloni et al. [13].

In the literature, relative standard deviations (RSD) of <5–10% [19,23,24] for either component concentration or mass in the cup are generally reported but can reach up to 20% [13]. The average relative standard deviation for the studied experimental set was 2.5%, with the highest RSD of 8.5%. In this work, the component masses in the cup were calculated by integration of the extraction kinetics curves with Equation (3). Hence, the error caused by the variation in the final EC mass in the cup was minimized.

### 3.3. Influencing the EC Component Mass in the Cup

#### 3.3.1. Linear Response Surfaces

The results from the experiments of the central composite design were evaluated using the response surface methodology (Section 2.8.3). The resulting linear regression parameters for the analyzed components and brew ratios are available in Table 3. The OriginPro file, including the utilized ANOVA tables, F-tests, and Pareto charts of standardized effects, can be found in Supplementary Materials S4.1 and S4.2.

Additionally, in Table 3, the adjusted coefficient of determination (adjusted R^2^) is included as an indicator of how well the fitted parameters describe the experimental data. The comparatively low coefficients of determination show that the response surface methodology can only partially explain the observed data variations. The adjusted coefficients of determination are particularly low for caffeine.

A quantitative interpretation, therefore, should be treated with care. Nevertheless, this study is the most comprehensive experimental study for extraction kinetics to date and yields important trends for trigonelline, caffeine, 5-CQA, and TSD mass in the EC cup. For quantitative analysis, mechanistic modelling should be considered. The trends for the analyzed parameters are discussed in the following Section 3.3.2, Section 3.3.3, Section 3.3.4 and Section 3.3.5 based on cross-sections of the response surface generated by Equation (4) with the coefficients $\beta_{m}$ (Table 3).

#### 3.3.2. Flow Rate Influence

**Figure 3:**
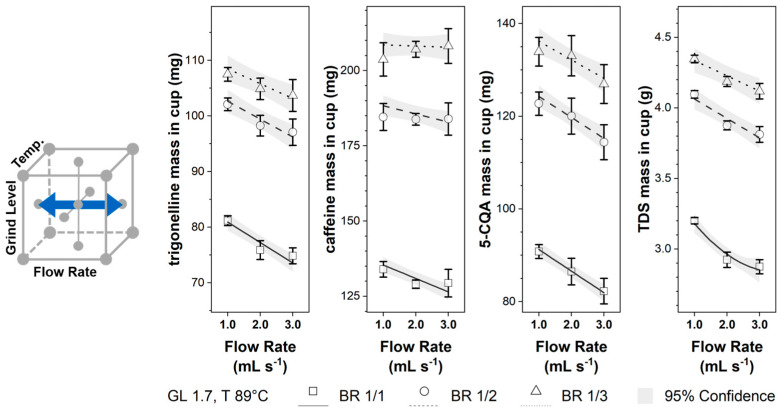
Trigonelline, caffeine, 5-CQA, and TDS masses in the EC cup for flow rates 1.0–3.0 mL s^−1^ and brew ratios BR 1/1, BR 1/2, and BR 1/3 at constant grinding level GL 1.7 and temperature T 89 °C; Lines: calculated data (Table 3) with 95% confidence band; □, o, ∆: experimental data (Table 2) with standard deviation.

As can be observed in Figure 3, increasing the flow rate reduced the mass in the cup for all components. The influence of the flow rate on the component mass in the cup was smallest for caffeine. While most components showed a similar trend (with respect to the slope) for the different brew ratios, the effect of flow rate on the caffeine concentration decreased from BR 1/1 to BR 1/3. In the literature, pressure control was preferred for the EC brewing process. Hence, no systematic study was found to compare the flow rate influence on the component mass in the cup. However, as postulated by Darcy’s Law, an increasing flow rate corresponded directly to an increasing brew pressure at otherwise constant conditions. As reported in the literature [22,23,24], increasing pressure reduces component masses in the cup, which correlates well with the observed reduction in trigonelline, caffeine, 5-CQA, and TDS masses in the cup by increasing flow rates. As observed in Section 3.1 for the beginning of the brew, a faster flow rate reduces the extract’s component concentration, which the time-dependent mass transport process can achieve.

#### 3.3.3. Grinding Level Influence

**Figure 4:**
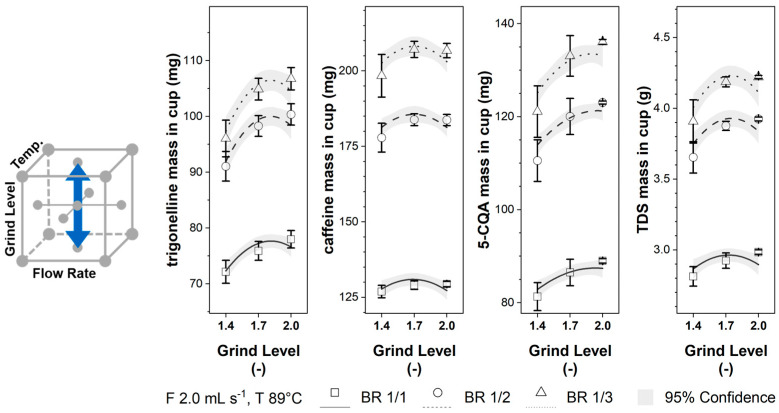
Trigonelline, caffeine, 5-CQA, and TDS masses in the EC cup for grinding levels 1.4–2.0 and brew ratios BR 1/1, BR 1/2, and BR 1/3 at constant flow rate F 2.0 mL s^−1^ and temperature T 89 °C; Lines: calculated data (Table 3) with 95% confidence band; □, o, ∆: experimental data (Table 2) with standard deviation.

For the analyzed grinding levels, the component masses in the cup for trigonelline, caffeine, 5-CQA, and TDS in Figure 4 show a parabolic behavior, as indicated by the significance of the coefficient $\beta_{5}$ (see Table 3). Changing the grinding level from GL 1.4 to GL 1.7 increases the component mass in the cup. Further increasing the grinding level to GL 2.0 results in a near-constant 5-CQA mass in the cup and decreases the component mass in the cup for trigonelline, caffeine, and TDS. Again, the caffeine mass in the cup is only slightly influenced.

In the literature, an increase in the extracted component mass in the cup is reported for smaller particles and commonly explained by the larger particle surface area [14,15,16,17]. The influence was described for ground coffee with significantly different particle sizes, often generated by sieving the coffee particles into fine and coarse fractions after grinding. The grinder settings used in this study resulted in more realistic, however, rather similar particle size distributions (see Appendix A Figure A1). The characteristic Sauter diameters for the particle size distributions were not significantly different for GL 1.4, GL 1.7, and GL 2.0; $d_{32}^{GL1.4}$ = 28.3 (SD 1.6), $d_{32}^{GL1.7}$ = 26.9 (SD 3.2) and $d_{32}^{GL2.0}$ = 29.2 (SD 1.4). The Sauter diameter is an integral measure of the particle size distribution, representing its specific surface area. Thus, the insignificance of difference in Sauter diameters indicates similar specific surface areas. Therefore, the explanation found in the literature, based on the particle surface area, cannot explain the observed influence of the grinding levels on the component masses in the cup in this study. It should, however, be noted that the minor differences in the particle size distributions for GL 1.4, GL 1.7, and GL 2.0 are already sufficient to influence the maximal brew pressure.

For similar EC grinding levels, Cameron et al. observed a reassembling influence on the extraction yield [43]. They attribute the decrease in the extraction yield for finer grinding levels to a possible partial clogging inside the coffee puck, which might decrease its permeability and increase the pressures needed to keep a set flow rate. The pressure measured during our experiments agrees with the hypothesis of possible clogging. Namely, the pressure was 3.8 bar, 7.4 bar, and 9.3 bar for the respective grinding levels GL 2.0, GL 1.7, and GL 1.4 and hence was higher for finer grinding.

#### 3.3.4. Temperature Influence

**Figure 5:**
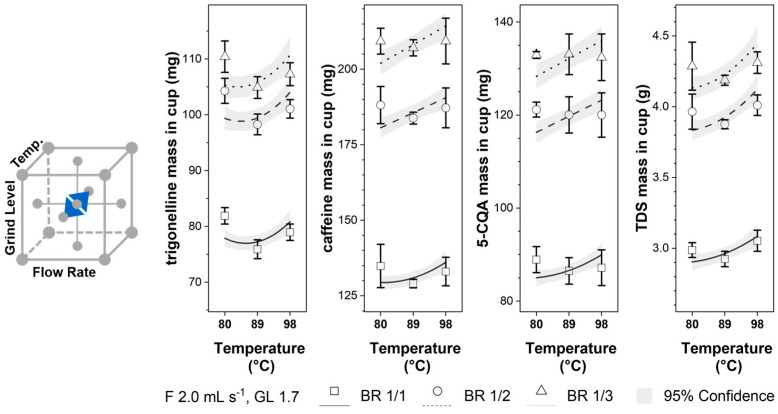
Trigonelline, caffeine, 5-CQA, and TDS masses in the EC cup for temperatures 80–98 °C and brew ratios BR 1/1, BR 1/2, and BR 1/3 at constant flow rate F 2.0 mL s^−1^ and grinding level GL 1.7; Lines: calculated data (Table 3) with 95% confidence band; □, o, ∆: experimental data (Table 2) with standard deviation.

The influence of the temperature variation from 80 °C to 98 °C is shown in Figure 5. As can be seen in Table 3, the quadratic coefficient $\beta_{6}$ is only significant for selected components and brew ratios yielding parabolic shapes. Generally, increasing temperature increases the component mass in the EC cup, which was not expected from the extraction kinetics presented in Section 3.1 for individually changing the temperature at the DoE central point.

As discussed in the introduction, literature knowledge on the effect of temperature is inconclusive. The literature reports different trends and only minor information to track potentially overlapping effects, which this study has attempted to avoid. However, note that the differences found in this study are significantly smaller than the values reported in the literature [13,29,30].

#### 3.3.5. Interactive Influences

The combined effects of the operating parameters are reflected in the parameters *β*_7_–*β*_9_ in Table 3. The combined effect of the grinding level and temperature is not significant for the investigated parameter space (*β*_9_ = 0). However, there are combined effects of the flow rate with the grinding level (*β*_7_) and the flow rate with the temperature (*β*_8_).

**Figure 6:**
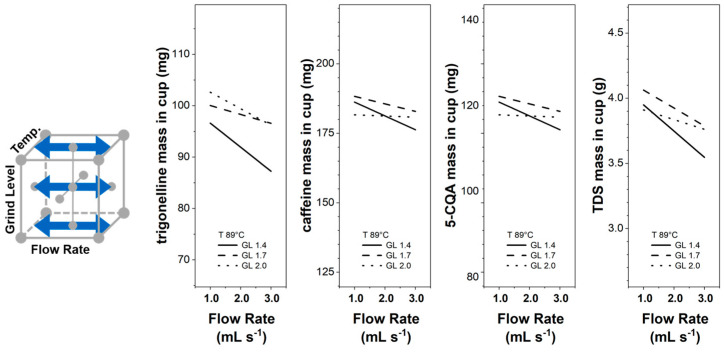
Trigonelline, caffeine, 5-CQA, and TDS masses in the EC cup for flow rates F 1.0–3.0 mL s^−1^ and grinding levels GL 1.4, GL 1.7, and GL 2.0 at temperatures 89 °C and brew ratio BR 1/2; 95% confidence bands omitted for clarity.

Figure 6 presents the influence of the flow rate on the component mass in the cup for three different grinding levels at BR 1/2 and T 89 °C. The flow rate increase has a higher effect on decreasing the component mass in the cup for the finer grinding level (GL 1.4) than for the coarser grinding levels. For different grinding levels, even though the particle size distribution was similar, the flow rate change resulted in different brew pressures.

For GL 1.4, the brew pressure increases from 2.9 bar for flow rate 1.0 mL s^−1^ (average pressure of Exp. 8 and Exp. 9 in Table 2) to 8.0 bar for flow rate 3.0 mL s^−1^ (Exp. 12, Exp. 13). For the same flow rate settings at GL 2.0, the pressure increases from 2.8 bar for flow rate 1.0 mL s^−1^ (Exp. 10, Exp. 11) to 3.6 bar for flow rate 3.0 mL s^−1^ (Exp. 14, Exp. 15). Higher pressures are known to decrease the component mass in the cup [22,23,24], which is in agreement with the experimental data in Table 2 and could explain the interaction between the influence of the flow rate and grinding level.

**Figure 7:**
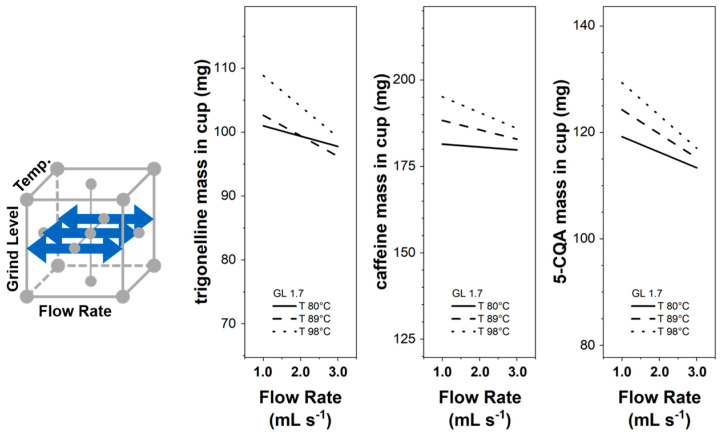
Trigonelline, caffeine, and 5-CQA masses in the EC cup for flow rates F 1.0–3.0 mL s^−1^ and temperatures 80 °C, 89 °C and 98 °C at grinding level GL 1.7 and brew ratio BR 1/2; 95% confidence bands omitted for clarity.

In Figure 7, the flow rate influence on the component mass in the cup is presented for three different temperatures at BR 1/2 and GL 1.7. The component mass in the cup decreases with an increase in the flow rate, whereas the influence is stronger at higher temperatures. This interaction could possibly explain the different temperature influences seen in Section 3.1 for the extraction kinetic curves and Section 3.3.4 resulting from the response surfaces. Temperature influences water viscosity and density [26], which might change uneven pressure and flow distributions in the coffee puck [25] and, in turn, influence the component mass transfer rate.

#### 3.3.6. Brew Ratio Influence

Overall, the effect of the parameters flow rate, grinding level, and temperature are small compared to the influences of different brew ratios. For example, decreasing the flow rate at the DoE central point (GL 1.7, T 89 °C) from 2.0 mL s^−1^ to 1.0 mL s^−1^ for the BR 1/2 changes the cup concentration of 5-CQA according to the response surface (see Table 3) from 2.99 mg g^−1^ to 3.11 mg g^−1^.

Using the extraction kinetics Equation (2), one can estimate the concentration in an EC cup if the specified brew ratio is experimentally not perfectly met. E.g. extracting 38 g instead of the intended 40 g (BR 1/2) already results in a concentration difference comparable to the one obtained by the above flow rate decrease from 2.0 mL s^−1^ to 1.0 mL s^−1^. While a 5% decrease in extracted mass appears to be larger, it corresponds to a decrease in extraction time of ~1 s. Hence, for practical applications to extract a consistent EC, the brew ratio is the first parameters to control with high accuracy before optimizing the process parameters flow rate, grinding level and water temperature.

## 4. Conclusions

The extraction kinetics for the espresso coffee (EC) components trigonelline, caffeine, 5-caffeoylquinic acid (5-CQA), and Total Dissolved Solids (TDS) were studied for water flow rates 1.0–3.0 mL s^−1^, grinding levels 1.4–2.0 (Mahlkoenig E65S), and water temperatures 80–98 °C. Through integration of the extraction kinetics, the component masses in the EC cup were determined for different brew rations. Based on a central composite design of experiment (DoE), the influence of the process parameters on the component masses in the cup for brew ratios BR 1/1, BR 1/2, and BR 1/3 were analyzed by response surface methodology. Comparably, low coefficients of variation allowed for qualitative rather than quantitative statements. For a quantitative assessment, the data-driven approach seems not to be entirely sufficient to capture the inherent complexity and irregularity of the coffee extraction process. Nevertheless, the following qualitative trends could be identified:

Trigonelline, 5-CQA, and TDS showed similar behaviors with respect to flow rate, grinding level, and temperature. Caffeine mass in the EC cup was only slightly influenced by different flow rates and grinding levels. Increasing the flow rate from 1.0 to 3.0 mL s^−1^ decreased the component masses in the cup. Despite the grinding level range of GL 1.4 to GL 2.0 leading to nearly identical particle size distributions, it still affected the brew pressure and component masses in the cup. In addition, finer grinding levels and higher temperatures increased the intensity of the flow rate influence on the component mass in the cup.

Overall, the experimental data showed good reproducibility and allowed interpretation of the extraction kinetics instead of only final concentrations in the EC cup. Especially, such kinetic data provide excellent grounds for further mechanistic modelling of the extraction process, potentially yielding a quantitative interpretation.

## Figures and Tables

**Table 1:** Face-centered Central Composite Design including axis, central (CP) and corner points with the parameter settings for flow rate (mL s^−1^), grinding level (−), and temperature (°C).

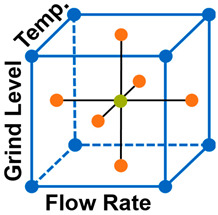		Experiment	Replicates	Flow Rate (mL s^−1^)	Grinding Level (−)	Temp. (°C)
**DoE Axis Points**	1	3	1.0	1.7	89
	2	3	3.0	1.7	89
	3	3	2.0	1.4	89
	4	3	2.0	2.0	89
	5	3	2.0	1.7	80
	6	3	2.0	1.7	98
**DoE CP**	7	6	2.0	1.7	89
**DoE Corner Points**	8	3	1.0	1.4	80
	9	3	1.0	1.4	98
	10	3	1.0	2.0	80
	11	3	1.0	2.0	98
	12	3	3.0	1.4	80
	13	3	3.0	1.4	98
	14	3	3.0	2.0	80
	15	3	3.0	2.0	98

**Table 2:** Trigonelline, caffeine, 5-CQA, and TDS masses in the EC cup for brew ratios BR 1/1, BR 1/2, and BR 1/3, set grinding levels, and the measured flow rates, temperatures, and pressures. The reported masses are averaged values with relative standard deviations in % provided in brackets.

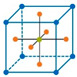	**Experiment**	**Flow Rate** **(mL s^−1^)**	**Grinding** **Level (−)**	**Temp.** **(°C)**	**Replicates**	**Mass in Cup; (RSD)**												**Pressure (Bar)**
						**Trigonelline (mg)**			**Caffeine (mg)**			**5-CQA (mg)**			**TDS (g)**			
						Brew Ratio			Brew Ratio			Brew Ratio			Brew Ratio			
						1/1	1/2	1/3	1/1	1/2	1/3	1/1	1/2	1/3	1/1	1/2	1/3	
DOE Axis Points	1	**1.0**	**1.7**	**87.3**	3	81.2	102.1	107.5	134.0	184.6	203.7	90.8	122.7	133.9	3.20	4.10	4.35	2.7
		(7.5)		(0.2)		(1.1)	(1.1)	(1.2)	(1.9)	(2.4)	(2.7)	(1.7)	(2.1)	(2.3)	(0.7)	(0.6)	(0.6)	(4.0)
	2	**2.8**	**1.7**	**88.2**	3	74.9	97.1	103.7	129.4	183.9	208.2	82.3	114.4	126.9	2.87	3.81	4.12	5.3
		(1.0)		(0.3)		(1.9)	(2.4)	(2.8)	(3.6)	(2.9)	(2.8)	(3.3)	(3.3)	(3.3)	(1.7)	(1.5)	(1.3)	(35.3)
	3	**1.9**	**1.4**	**88.5**	3	72.2	91.1	96.0	126.9	177.9	198.4	81.3	110.5	121.1	2.81	3.65	3.91	3.9
		(0.3)		(0.1)		(2.8)	(2.9)	(3.4)	(1.6)	(2.7)	(3.6)	(3.7)	(4.1)	(4.6)	(2.4)	(3.0)	(3.9)	(5.1)
	4	**2.0**	**2.0**	**88.6**	3	78.0	100.3	106.7	129.4	183.7	206.7	89.0	123.0	136.1	2.98	3.92	4.22	3.3
		(0.2)		(0.1)		(2.0)	(1.9)	(1.9)	(0.7)	(1.0)	(1.1)	(0.5)	(0.3)	(0.4)	(0.5)	(0.4)	(0.3)	(3.4)
	5	**1.9**	**1.7**	**79.2**	3	81.9	104.3	110.4	134.8	188.2	209.3	88.9	121.2	132.9	2.99	3.96	4.29	3.6
		(0.7)		(0.1)		(1.8)	(2.1)	(2.6)	(5.3)	(3.3)	(2.0)	(3.1)	(1.3)	(0.6)	(1.7)	(3.1)	(4.0)	(7.7)
	6	**1.9**	**1.7**	**96.7**	3	78.9	101.1	107.3	133.0	187.2	209.3	87.2	120.0	132.4	3.05	4.01	4.31	3.0
		(1.0)		(0.2)		(1.9)	(1.6)	(1.9)	(3.5)	(3.5)	(3.6)	(4.4)	(4.0)	(3.8)	(2.4)	(1.8)	(1.8)	(4.6)
DoE Central Point	7	**1.9**	**1.7**	**88.3**	6	75.9	98.3	104.9	129.0	183.8	207.1	86.5	120.0	133.1	2.92	3.88	4.19	3.4
		(2.3)		(0.5)		(2.2)	(1.9)	(1.8)	(1.1)	(1.1)	(1.3)	(3.3)	(3.2)	(3.3)	(1.9)	(0.8)	(0.8)	(15.6)
DOE Corner Points	8	**1.0**	**1.4**	**78.8**	3	78.2	95.3	99.0	132.1	177.8	193.6	86.7	114.0	122.6	3.15	3.84	3.99	2.9
		(0.1)		(0.1)		(0.7)	(1.5)	(1.9)	(2.2)	(2.0)	(2.0)	(0.5)	(0.5)	(0.9)	(4.6)	(1.1)	(0.7)	(3.6)
	9	**0.9**	**1.4**	**96.2**	3	80.1	102.2	108.4	139.8	195.2	217.2	92.6	126.4	138.8	3.22	4.18	4.46	2.8
		(2.3)		(0.2)		(5.0)	(5.6)	(6.0)	(4.2)	(5.1)	(5.8)	(3.5)	(4.4)	(5.0)	(3.9)	(4.7)	(5.1)	(4.3)
	10	**1.0**	**2.0**	**78.7**	3	78.0	96.6	101.1	127.0	172.9	189.6	87.5	117.0	126.9	2.92	3.72	3.94	2.9
		(0.9)		(0.1)		(4.1)	(7.3)	(8.5)	(3.6)	(6.0)	(7.4)	(3.9)	(6.2)	(7.4)	(3.7)	(6.5)	(7.7)	(3.8)
	11	**0.9**	**2.0**	**96.1**	3	83.9	106.1	111.9	135.3	187.8	208.2	94.9	129.0	141.3	3.19	4.11	4.38	2.6
		(5.9)		(0.3)		(0.8)	(1.4)	(1.6)	(0.6)	(1.0)	(1.3)	(1.6)	(2.2)	(2.5)	(1.0)	(0.6)	(1.0)	(2.3)
	12	**2.7**	**1.4**	**79.8**	3	69.0	88.3	93.8	120.4	172.4	195.0	77.1	106.9	118.4	2.67	3.53	3.81	8.4
		(3.7)		(1.0)		(4.3)	(2.9)	(2.2)	(5.7)	(4.3)	(3.4)	(4.3)	(3.1)	(2.4)	(4.3)	(2.0)	(0.9)	(16.7)
	13	**2.7**	**1.4**	**97.1**	3	72.8	93.2	99.0	129.2	184.0	207.2	83.0	114.4	126.3	2.89	3.83	4.13	7.6
		(2.3)		(0.4)		(2.2)	(2.0)	(2.5)	(1.8)	(1.5)	(2.0)	(2.7)	(2.3)	(2.4)	(2.3)	(1.9)	(2.5)	(9.3)
	14	**2.9**	**2.0**	**79.1**	3	75.1	97.8	104.7	122.6	176.3	199.8	81.8	115.5	128.7	2.75	3.68	3.99	3.6
		(0.8)		(0.1)		(2.9)	(1.9)	(1.5)	(3.4)	(2.8)	(2.5)	(0.5)	(0.7)	(0.2)	(1.3)	(0.9)	(1.0)	(7.4)
	15	**2.8**	**2.0**	**96.4**	3	76.2	98.9	105.7	127.5	182.7	206.6	86.9	120.7	131.4	2.93	3.88	4.19	3.5
		(8.0)		(0.5)		(1.7)	(1.0)	(0.8)	(1.7)	(1.9)	(2.1)	(1.9)	(1.5)	(1.6)	(0.8)	(0.9)	(1.0)	(7.9)
	**Average mass in cup:**					**77.1**	**98.2**	**104.0**	**130.0**	**182.6**	**204.0**	**86.4**	**118.4**	**130.1**	**2.97**	**3.87**	**4.15**	

**Table 3:** Linear regressions and respective adjusted R^2^, derived by response surface methodology for flow rates [1.0 mL s^−1^, 3.0 mL s^−1^], grinding levels [1.4, 2.0], and temperatures [80 °C, 98 °C] to calculate the component masses in the EC cup for trigonelline, caffeine, 5-CQA, and TDS at brew ratios BR 1/1, BR 1/2, and BR 1/3.

$m_{cup}=\beta_{0}+\beta_{1}x_{flow}+\beta_{2}x_{grind}+\beta_{3}x_{temp}+\beta_{4}x_{flow}^{2}+\beta_{5}x_{grind}^{2}+\beta_{6}x_{temp}^{2}+\beta_{7}x_{flow}x_{grind}+\beta_{8}x_{flow}x_{temp}+\beta_{9}x_{grind}x_{temp}$												
Components	Brew Ratio	$\beta_{0}$ (mg)	$\beta_{1}$ (mg s mL^−1^)	$\beta_{2}$ (mg)	$\beta_{3}$ (mg °C^−1^)	$\beta_{4}$ (mg s^2^ mL^−2^)	$\beta_{5}$ (mg)	$\beta_{6}$ (mg °C^−2^)	$\beta_{7}$ (mg s mL^−1^)	$\beta_{8}$ (mg °C^−1^ s mL^−1^)	$\beta_{9}$ (mg °C^−1^)	R^2^ (adj.)
**Trigonelline**	**1/1**	185.8	−9.17	105.90	−4.48	0	−30.96	0.03	3.24	0	0	0.66
	**1/2**	134.6	4.28	163.33	−4.40	0	−47.80	0.03	4.92	−0.18	0	0.60
	**1/3**	99.9	8.54	184.84	−4.06	0	−54.17	0.03	5.71	−0.24	0	0.58
**Caffeine**	**1/1**	192.0	−13.85	118.83	−3.59	0	−38.45	0.02	5.52	0	0	0.50
	**1/2**	−9.2	2.92	149.27	0.97	0	−48.37	0	7.57	−0.21	0	0.41
	**1/3**	−57.7	15.87	186.29	1.37	0	−59.53	0	8.42	−0.34	0	0.42
**5-CQA**	**1/1**	112.2	−10.32	56.75	−1.74	0	−16.42	0.01	3.33	0	0	0.69
	**1/2**	−10.4	2.96	83.84	0.74	0	−24.01	0	4.97	−0.18	0	0.62
	**1/3**	−54.7	13.94	119.15	1.02	0	−34.02	0	4.93	−0.30	0	0.57
		(g)	(g s mL^−1^)	(g)	(g °C^−1^)	(g s^2^ mL^−2^)	(g)	(g °C^−2^)	(g s mL^−1^)	(g °C^−1^ s mL^−1^)	(g °C^−1^)	
**TDS**	**1/1**	3.65	−0.70	2.78	−0.06	0.05	−0.91	0.001	0.19	0	0	0.75
	**1/2**	4.19	−0.50	4.75	−0.10	0	−1.48	0.001	0.21	0	0	0.64
	**1/3**	2.91	0.12	6.16	−0.11	0	−1.87	0.001	0.20	−0.01	0	0.57

## Data Availability

The data used to support the findings of this study can be made available by the corresponding author upon request.

## Supplementary Materials

The following supporting information can be downloaded at: https://www.mdpi.com/article/10.3390/foods12152871/s1, Table S1: Experiment raw data; Table S2: Extraction kinetic fitting parameters for single experiments; Table S3: Component mass in the cup for single experiments; S4.1: OriginPro file; S4.2: Summary of OriginPro fitting and evaluation calculations.

## Appendix A

**Table A1:** Parameter values with standard error (SE) for the experiments’ average extraction kinetics $c(m_{\sum })$ for trigonelline, caffeine, 5-CQA, and TDS, based on Equation (2).

Experiment	*c*(*m*_∑_) = *c*_0_ exp(−*m*_∑_/*λ*)											
	Trigonelline						Caffeine					
	*c*_0_ (mg g^−1^)		*λ* (g)		Statistics		*c*_0_ (mg g^−1^)		*λ* (g)		Statistics	
	Value	SE	Value	SE	Red. Chi-Sqr	Adj. R^2^	Value	SE	Value	SE	Red. Chi-Sqr	Adj. R^2^
1	7.40899	0.12316	14.76344	0.42552	0.02561	0.99481	10.4887	0.14327	20.52467	0.5199	0.04752	0.99493
2	6.46932	0.23064	16.40303	0.86193	0.05312	0.9806	9.46765	0.28924	23.93518	1.18507	0.13117	0.97824
3	6.54716	0.23705	14.93682	0.84702	0.06872	0.97702	9.66427	0.27442	21.96458	1.0845	0.14334	0.97831
4	6.83473	0.11231	15.99516	0.42845	0.01714	0.99521	9.67546	0.15595	23.05736	0.66335	0.04944	0.99303
5	7.30706	0.16914	15.42548	0.57294	0.03804	0.9907	10.38155	0.30436	21.59864	1.08663	0.174	0.97851
6	6.95468	0.10214	15.7859	0.3698	0.01422	0.99616	10.04235	0.15921	22.39093	0.62077	0.05039	0.99327
7	6.69949	0.09587	16.08737	0.36681	2.47 × 10^−2^	0.99227	9.70981	0.13064	23.09434	0.5462	0.06781	0.9897
8	7.62541	0.09065	13.12089	0.27877	0.01422	0.9973	10.69172	0.15672	18.92703	0.52514	0.0595	0.99409
9	7.08449	0.1493	15.63306	0.56872	0.03722	0.99154	10.69195	0.19535	21.67685	0.73662	0.08821	0.99072
10	7.34872	0.18011	14.07208	0.54291	0.03783	0.99095	10.13843	0.25125	19.75409	0.82583	0.10739	0.98646
11	7.57984	0.13439	15.04329	0.43899	0.02663	0.99434	10.45723	0.1349	21.14279	0.4897	0.03828	0.99552
12	6.17348	0.332	15.48473	1.32764	0.13618	0.94866	8.97304	0.39549	23.57685	1.85949	0.3117	0.94656
13	6.50908	0.26715	15.53493	1.03374	0.09268	0.97024	9.67713	0.31126	23.15069	1.31948	0.19488	0.97264
14	6.47069	0.1751	16.63498	0.70499	0.03894	0.98677	9.03485	0.21274	24.1222	0.98821	0.08722	0.98465
15	6.62197	0.14144	16.37875	0.56426	0.02867	0.9911	9.45174	0.17933	23.78621	0.80464	0.06779	0.98933
**Experiment**	**5-CQA**						**TDS**					
	***c*_0_ (mg g^−1^)**		***λ* (g)**		**Statistics**		***c*_0_ (g g^−1^)**		***λ* (g)**		**Statistics**	
	**Value**	**SE**	**Value**	**SE**	**Red.** **Chi-Sqr**	**Adj. R^2^**	**Value**	**SE**	**Value**	**SE**	**Red.** **Chi-Sqr**	**Adj. R^2^**
1	7.32493	0.1108	19.11304	0.52774	0.02668	0.99429	0.28305	0.00386	15.70033	0.37567	2.68 × 10^−5^	0.9962
2	6.35357	0.22661	21.1787	1.18384	0.07051	0.97413	0.23924	0.00955	17.83024	1.06401	1.02 × 10^−4^	0.97201
3	6.47778	0.22434	19.62703	1.14453	0.08511	0.97164	0.24196	0.00933	16.59384	1.03039	1.21 × 10^−4^	0.97078
4	6.92748	0.11967	20.80018	0.62438	0.02622	0.99288	0.25164	0.00514	17.31233	0.58753	3.94 × 10^−5^	0.99185
5	7.08289	0.1861	19.75796	0.87311	0.05984	0.98429	0.2474	0.00636	17.98499	0.75292	6.37 × 10^−5^	0.98585
6	6.80018	0.12522	20.60333	0.6479	0.02871	0.9918	0.25713	0.00367	17.3144	0.40346	2.05 × 10^−5^	0.99593
7	6.79246	0.10682	20.77137	0.55683	4.08 × 10^−2^	0.98752	0.24827	0.00419	17.47261	0.48029	5.20 × 10^−5^	0.98811
8	7.31745	0.09572	17.29798	0.4219	0.02055	0.99572	0.30725	0.00999	13.16232	0.76497	1.73 × 10^−4^	0.97956
9	7.32619	0.12423	19.92738	0.61563	0.03302	0.99274	0.27778	0.00462	16.50264	0.47902	3.77 × 10^−5^	0.99428
10	7.18869	0.17832	18.48345	0.76212	0.05056	0.98742	0.2599	0.00608	15.62012	0.58694	4.89 × 10^−5^	0.99069
11	7.57346	0.10943	19.56499	0.49683	0.02338	0.99488	0.27975	0.00446	16.03038	0.42671	3.15 × 10^−5^	0.99499
12	6.02455	0.29418	20.79431	1.75317	0.15129	0.94255	0.22578	0.01287	17.38228	1.62994	2.36 × 10^−4^	0.93514
13	6.53399	0.25585	20.40671	1.37513	0.11588	0.96435	0.24308	0.01083	17.58935	1.30555	1.76 × 10^−4^	0.96041
14	6.35075	0.15431	21.56882	0.88151	0.04089	0.98564	0.22567	0.00635	18.4123	0.83369	5.79 × 10^−5^	0.98403
15	6.76424	0.14962	21.01016	0.79908	0.04192	0.98738	0.24533	0.00629	17.69504	0.74554	6.18 × 10^−5^	0.98599
