# Supplementary material for: Influence of Flow Rate, Particle Size, and Temperature on Espresso Extraction Kinetics
Source: Foods. 2023 Jul 28;12(15):2871. doi: 10.3390/foods12152871 (PMC10418593; doi:10.3390/foods12152871)

Response Surface Fit by OriginPro

This file contains the result sheets of the response surface fittings based on the reduced linear regression for the components trigonelline, caffeine, 5-caffeoylquinic acid (V-CQA) and Total Dissolved Solids (TDS) for Ristretto (Ris, brew ration 1/1), Espresso (Esp, brew ratio 1/2) and Espresso Lungo (Lungo, brew ration 1/3). The response surface is based on the factors flow rate(A) with a range from 1.0 mL s^{-1} to 3.0 mL s^{-1}, grinding level (B) with a range from 1.4 to 2.0 and temperature (C) from 80 °C to 98 °C. For the evaluation in OriginPro the factor ranges were translated to the interval [-1, 1]. The following result sheets include the coded coefficients for the response surface, R^2 statistics and ANOVA as well as the fitted plot, residual plots, effect plots and main/interaction plots.

Table of Content:

1. Trigonelline response surface fit
   1.1. for ristretto 1
   1.2. for espresso 2
   1.3. for espresso lungo 3
2. Caffeine
   2.1. for ristretto 4
   2.2. for espresso 5
   2.3. for espresso lungo 6
3. 5-caffeoylquinic acid (V-CQA)
   3.1. for ristretto 7
   3.2. for espresso 8
   3.3. for espresso lungo 9
4. Total Dissolved Solids (TDS)
   4.1. for ristretto 10
   4.2. for espresso 11
   4.3. for espresso lungo 12

1.1 Trigonelline - Ristretto

Coded Coefficients

	Value	Standard Error	95% LCL	95% UCL	t-Value	Prob > |t|
(Intercept)	77.25365	0.62006	76.00142	78.50589	224.06896	5.96466E-65
A	-3.6636	0.47169	-4.6162	-2.71099	-10.62601	2.39635E-13
B	2.13968	0.4408	1.24947	3.0299	6.20601	2.20316E-7
C	1.48397	0.50387	0.46638	2.50155	4.30414	1.016E-4
B*B	-2.78605	0.79483	-4.39124	-1.18085	-8.08073	5.13877E-10
C*C	2.11142	0.83376	0.42761	3.79522	6.12402	2.88199E-7
A*B	0.97216	0.52814	-0.09444	2.03875	2.81968	0.00737

Statistics

	Trigonellin_mass_in_Ristretto
Degress of Freedom	41
Root Mean Square of Error	2.38868
R-Square	0.70652
Adj. R-Square	0.66358
Residual Sums of Squares	233.93747
Predicted. R-Square	0.5923

ANOVA

	DF	Sum of Squares	Mean Square	F Value	Prob>F
A	1	322.40718	322.40718	56.50525	3.09963E-9
B	1	124.86871	124.86871	21.88455	3.13869E-5
C	1	21.45691	21.45691	3.76055	0.05938
B*B	1	38.60104	38.60104	6.76524	0.01287
C*C	1	36.52088	36.52088	6.40067	0.01534
A*B	1	19.33286	19.33286	3.38829	0.07291
Error	41	233.93747	5.70579		
Total	47	797.12505			

Fitted Plot

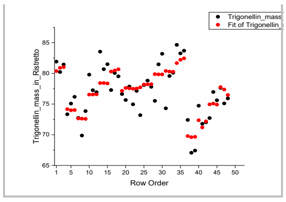

Residual Plot

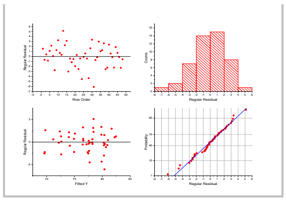

Effects Plot

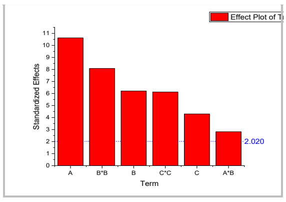

Main Effects Plot

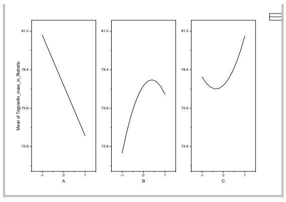

2-way Interaction Plot

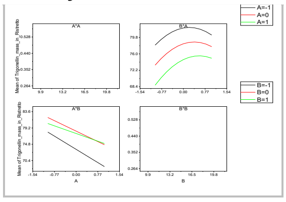

1.2 Trigonelline - Espresso

Coded Coefficients

	Value	Standard Error	95% LCL	95% UCL	t-Value	Prob > |t|
(Intercept)	99.42477	0.84951	97.70785	101.1417	210.65853	1.52209E-62
A	-3.20914	0.65288	-4.52865	-1.88963	-6.79944	3.57107E-8
B	3.1912	0.60441	1.96965	4.41275	6.76144	4.03563E-8
C	2.33129	0.69319	0.9303	3.73228	4.93948	1.43558E-5
B*B	-4.30238	1.08902	-6.50338	-2.10138	-9.11577	2.5996E-11
C*C	2.2806	1.14561	-0.03476	4.59596	4.83208	2.01876E-5
A*B	1.4759	0.72379	0.01307	2.93873	3.12711	0.00329
A*C	-1.60313	0.7496	-3.11813	-0.08813	-3.39667	0.00155

Statistics

	Trigonellin_mass_in_Espresso
Degress of Freedom	40
Root Mean Square of Error	3.26991
R-Square	0.66052
Adj. R-Square	0.60111
Residual Sums of Squares	427.69322
Predicted. R-Square	0.48749

ANOVA

	DF	Sum of Squares	Mean Square	F Value	Prob>F
A	1	197.9553	197.9553	18.51377	1.05685E-4
B	1	284.78395	284.78395	26.63441	7.08211E-6
C	1	96.55504	96.55504	9.03031	0.00457
B*B	1	119.82019	119.82019	11.20618	0.00178
C*C	1	35.04261	35.04261	3.27736	0.07776
A*B	1	49.08811	49.08811	4.59096	0.03828
A*C	1	48.90461	48.90461	4.5738	0.03862
Error	40	427.69322	10.69233		
Total	47	1259.84304			

Fitted Plot

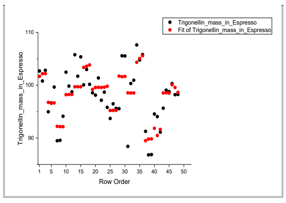

Residual Plot

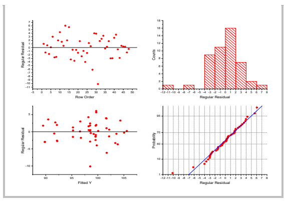

Effects Plot

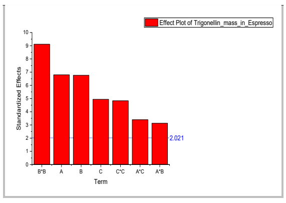

Main Effects Plot

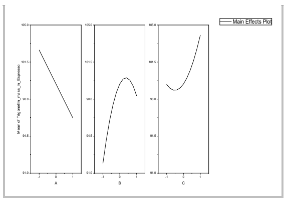

2-way Interaction Plot

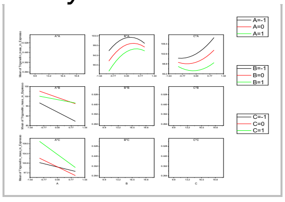

1.3 Trigonelline - Espresso Lungo

Coded Coefficients

	Value	Standard Error	95% LCL	95% UCL	t-Value	Prob > |t|
(Intercept)	105.80572	0.96089	103.8637	107.74774	198.19396	1.74401E-61
A	-2.66583	0.73847	-4.15834	-1.17332	-4.9936	1.20841E-5
B	3.62049	0.68365	2.23878	5.00219	6.78185	3.77902E-8
C	2.68034	0.78407	1.09567	4.26501	5.02078	1.10811E-5
B*B	-4.8757	1.2318	-7.36526	-2.38614	-9.1331	2.46816E-11
C*C	2.19587	1.2958	-0.42305	4.81479	4.11328	1.88745E-4
A*B	1.71388	0.81868	0.05926	3.36849	3.21041	0.00261
A*C	-2.11514	0.84788	-3.82876	-0.40151	-3.96205	2.98058E-4

Statistics

	Trigonellin_mass_in_Lungo
Degress of Freedom	40
Root Mean Square of Error	3.69862
R-Square	0.64106
Adj. R-Square	0.57824
Residual Sums of Squares	547.19063
Predicted. R-Square	0.45445

ANOVA

	DF	Sum of Squares	Mean Square	F Value	Prob>F
A	1	115.30154	115.30154	8.42862	0.00598
B	1	366.30049	366.30049	26.77681	6.77638E-6
C	1	145.92848	145.92848	10.66747	0.00224
B*B	1	167.49235	167.49235	12.2438	0.00116
C*C	1	30.05441	30.05441	2.197	0.14612
A*B	1	67.04872	67.04872	4.90131	0.0326
A*C	1	85.13146	85.13146	6.22317	0.01684
Error	40	547.19063	13.67977		
Total	47	1524.44808			

Fitted Plot

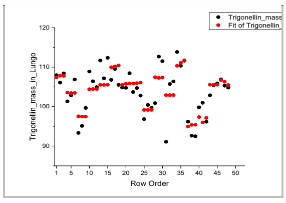

Residual Plot

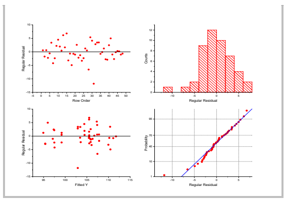

Effects Plot

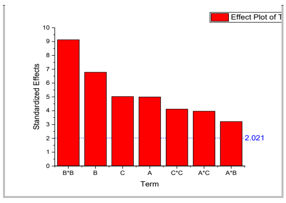

Main Effects Plot

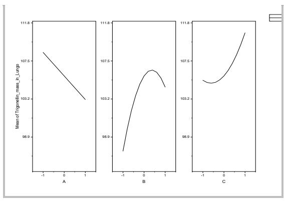

2-way Interaction Plot

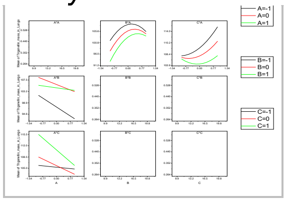

2.1 Caffeine - Ristretto

Coded Coefficients

	Value	Standard Error	95% LCL	95% UCL	t-Value	Prob > |t|
(Intercept)	130.92848	1.05251	128.8029	133.05406	223.71955	6.35867E-65
A	-4.46659	0.80067	-6.08357	-2.8496	-7.63213	2.14247E-9
B	-0.26118	0.74823	-1.77227	1.24991	-0.44628	0.65774
C	3.36105	0.85529	1.63376	5.08834	5.74308	1.00324E-6
B*B	-3.46066	1.34918	-6.18538	-0.73595	-5.91329	5.74752E-7
C*C	1.80262	1.41525	-1.05553	4.66077	3.08017	0.00369
A*B	1.65553	0.89648	-0.15494	3.46601	2.82883	0.0072

Statistics

	Caffeine_mass_in_Ristretto
Degress of Freedom	41
Root Mean Square of Error	4.05463
R-Square	0.56785
Adj. R-Square	0.50461
Residual Sums of Squares	674.03971
Predicted. R-Square	0.40249

ANOVA

	DF	Sum of Squares	Mean Square	F Value	Prob>F
A	1	497.86186	497.86186	30.28358	2.19685E-6
B	1	6.72072	6.72072	0.4088	0.52613
C	1	220.79343	220.79343	13.43026	7.0356E-4
B*B	1	77.6838	77.6838	4.72529	0.03555
C*C	1	26.56816	26.56816	1.61607	0.21081
A*B	1	56.06556	56.06556	3.41032	0.07202
Error	41	674.03971	16.43999		
Total	47	1559.73324			

Fitted Plot

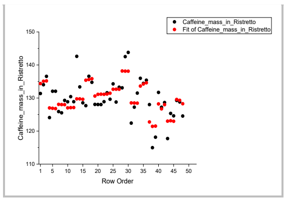

Residual Plot

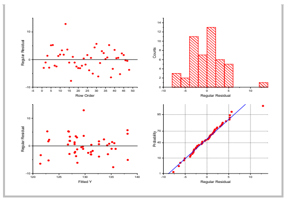

Effects Plot

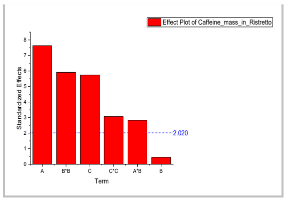

Main Effects Plot

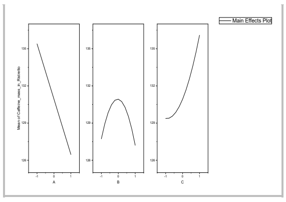

2-way Interaction Plot

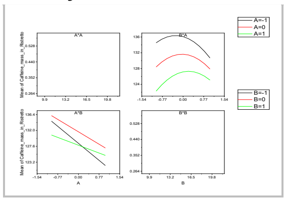

2.2 Caffeine - Espresso

Coded Coefficients

	Value	Standard Error	95% LCL	95% UCL	t-Value	Prob > |t|
(Intercept)	185.61236	1.35352	182.87886	188.34586	225.44543	4.64046E-65
A	-2.70581	1.13856	-5.00517	-0.40644	-3.28648	0.00208
B	-0.01655	1.05387	-2.14488	2.11177	-0.02011	0.98406
C	4.9825	1.0906	2.77999	7.185	6.05176	3.65184E-7
B*B	-4.35365	1.70167	-7.79025	-0.91705	-5.28796	4.4223E-6
A*B	2.27177	1.26258	-0.27807	4.8216	2.7593	0.00862
A*C	-1.87045	1.30275	-4.50141	0.76051	-2.27185	0.02841

Statistics

	Caffeine_mass_in_Espresso
Degress of Freedom	41
Root Mean Square of Error	5.70409
R-Square	0.48432
Adj. R-Square	0.40885
Residual Sums of Squares	1334.00052
Predicted. R-Square	0.26355

ANOVA

	DF	Sum of Squares	Mean Square	F Value	Prob>F
A	1	132.19427	132.19427	4.06294	0.05042
B	1	2.52931	2.52931	0.07774	0.78179
C	1	735.01171	735.01171	22.59031	2.47408E-5
B*B	1	202.37838	202.37838	6.22002	0.01676
A*B	1	113.67839	113.67839	3.49386	0.06875
A*C	1	67.07184	67.07184	2.06143	0.15865
Error	41	1334.00052	32.5366		
Total	47	2586.86442			

Fitted Plot

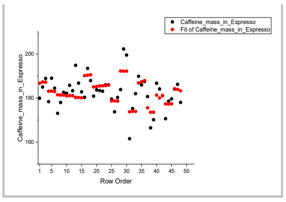

Residual Plot

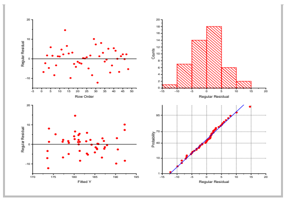

Effects Plot

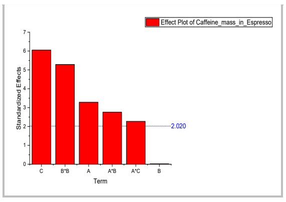

Main Effects Plot

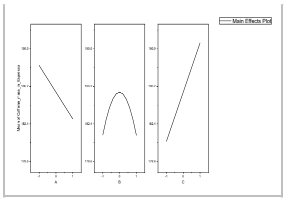

2-way Interaction Plot

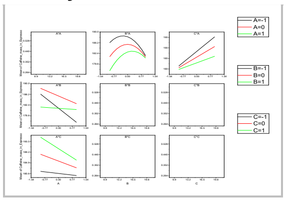

2.3 Caffeine - Espresso Lungo

Coded Coefficients

	Value	Standard Error	95% LCL	95% UCL	t-Value	Prob > |t|
(Intercept)	208.17047	1.60437	204.93037	211.41056	213.31173	4.48096E-64
A	-0.38506	1.34957	-3.11056	2.34044	-0.39457	0.69521
B	0.21677	1.24918	-2.306	2.73955	0.22213	0.82532
C	6.15691	1.29272	3.54621	8.7676	6.30897	1.57247E-7
B*B	-5.35806	2.01704	-9.43156	-1.28455	-5.49039	2.28944E-6
A*B	2.52729	1.49657	-0.4951	5.54968	2.58971	0.01324
A*C	-3.09214	1.54419	-6.21069	0.02642	-3.16851	0.00289

Statistics

	Caffeine_mass_in_Lungo
Degress of Freedom	41
Root Mean Square of Error	6.76122
R-Square	0.48987
Adj. R-Square	0.41522
Residual Sums of Squares	1874.2773
Predicted. R-Square	0.26046

ANOVA

	DF	Sum of Squares	Mean Square	F Value	Prob>F
A	1	1.86271	1.86271	0.04075	0.84103
B	1	0.11445	0.11445	0.0025	0.96034
C	1	1161.68375	1161.68375	25.41195	9.82833E-6
B*B	1	307.20602	307.20602	6.72016	0.01315
A*B	1	145.69842	145.69842	3.18717	0.08162
A*C	1	183.30158	183.30158	4.00974	0.05188
Error	41	1874.2773	45.71408		
Total	47	3674.14423			

Fitted Plot

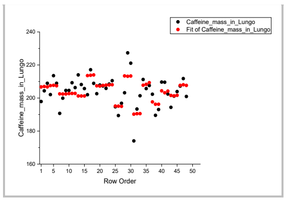

Residual Plot

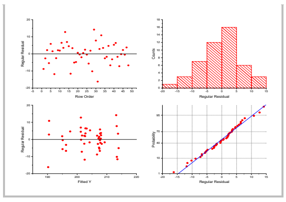

Effects Plot

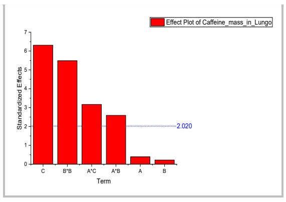

Main Effects Plot

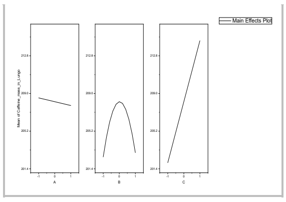

2-way Interaction Plot

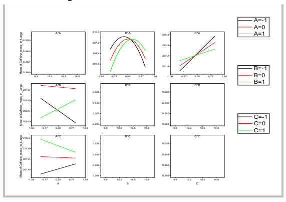

3.1 5-CQA - Ristretto

Coded Coefficients

	Value	Standard Error	95% LCL	95% UCL	t-Value	Prob > |t|
(Intercept)	86.58208	0.70892	85.15038	88.01377	219.64755	1.35008E-64
A	-4.66192	0.53929	-5.75105	-3.5728	-11.82669	8.54728E-15
B	2.27386	0.50397	1.25606	3.29166	5.76849	9.23222E-7
C	2.46971	0.57608	1.30629	3.63313	6.26534	1.814E-7
B*B	-1.47767	0.90874	-3.31291	0.35757	-3.74866	5.49275E-4
C*C	0.91814	0.95324	-1.00698	2.84325	2.3292	0.02486
A*B	0.99767	0.60383	-0.22178	2.21712	2.53097	0.01531

Statistics

	V_CQA_mass_in_Ristretto
Degress of Freedom	41
Root Mean Square of Error	2.731
R-Square	0.73098
Adj. R-Square	0.69162
Residual Sums of Squares	305.79357
Predicted. R-Square	0.63676

ANOVA

	DF	Sum of Squares	Mean Square	F Value	Prob>F
A	1	521.48022	521.48022	69.9187	2.12244E-10
B	1	136.47952	136.47952	18.29882	1.10281E-4
C	1	133.38332	133.38332	17.88369	1.28256E-4
B*B	1	12.32795	12.32795	1.6529	0.20578
C*C	1	6.88746	6.88746	0.92345	0.3422
A*B	1	20.36093	20.36093	2.72994	0.10612
Error	41	305.79357	7.45838		
Total	47	1136.71297			

Fitted Plot

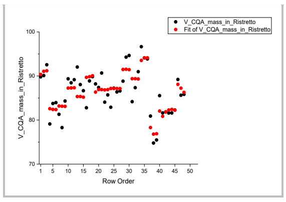

Residual Plot

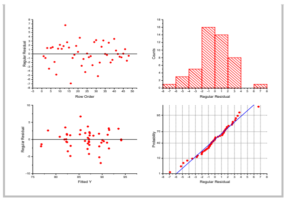

Effects Plot

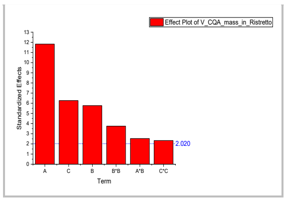

Main Effects Plot

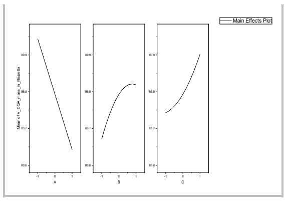

2-way Interaction Plot

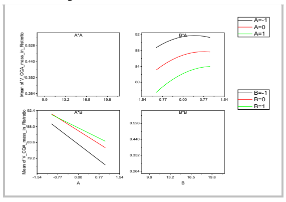

3.2 5-CQA - Espresso

Coded Coefficients

	Value	Standard Error	95% LCL	95% UCL	t-Value	Prob > |t|
(Intercept)	119.74944	0.93417	117.86285	121.63603	210.74146	7.36455E-64
A	-4.52782	0.7858	-6.11478	-2.94086	-7.96829	7.33663E-10
B	3.64304	0.72735	2.17413	5.11196	6.41122	1.12512E-7
C	3.45763	0.7527	1.93752	4.97774	6.08492	3.27592E-7
B*B	-2.161	1.17445	-4.53285	0.21085	-3.80304	4.67343E-4
A*B	1.49069	0.8714	-0.26913	3.25052	2.6234	0.01217
A*C	-1.61154	0.89912	-3.42736	0.20428	-2.83607	0.00706

Statistics

	V_CQA_mass_in_Espresso
Degress of Freedom	41
Root Mean Square of Error	3.93681
R-Square	0.66964
Adj. R-Square	0.62129
Residual Sums of Squares	635.4364
Predicted. R-Square	0.55179

ANOVA

	DF	Sum of Squares	Mean Square	F Value	Prob>F
A	1	415.2352	415.2352	26.79205	6.35428E-6
B	1	361.37871	361.37871	23.31709	1.94228E-5
C	1	362.67638	362.67638	23.40082	1.88924E-5
B*B	1	48.88507	48.88507	3.15419	0.08315
A*B	1	50.06604	50.06604	3.23039	0.07965
A*C	1	49.78851	49.78851	3.21248	0.08046
Error	41	635.4364	15.49845		
Total	47	1923.46631			

Fitted Plot

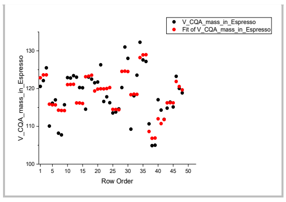

Residual Plot

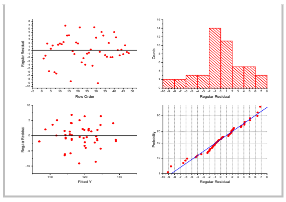

Effects Plot

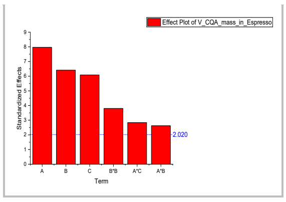

Main Effects Plot

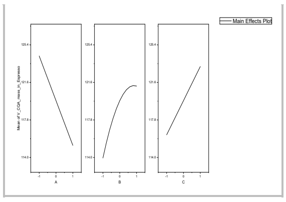

2-way Interaction Plot

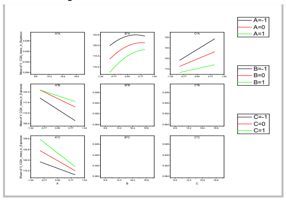

3.3 5-CQA - Espresso Lungo

Coded Coefficients

	Value	Standard Error	95% LCL	95% UCL	t-Value	Prob > |t|
(Intercept)	132.18312	1.11655	129.92821	134.43803	194.62529	1.91887E-62
A	-3.98835	0.93922	-5.88514	-2.09156	-5.87241	6.57066E-7
B	4.00511	0.86936	2.24941	5.76081	5.89709	6.06068E-7
C	3.84388	0.89965	2.02699	5.66077	5.65969	1.31758E-6
B*B	-3.06137	1.40374	-5.89629	-0.22645	-4.50753	5.38022E-5
A*B	1.47751	1.04153	-0.6259	3.58092	2.17547	0.03541
A*C	-2.65921	1.07466	-4.82954	-0.48888	-3.91539	3.33814E-4

Statistics

	V_CQA_mass_in_Lungo
Degress of Freedom	41
Root Mean Square of Error	4.70541
R-Square	0.62163
Adj. R-Square	0.56626
Residual Sums of Squares	907.77565
Predicted. R-Square	0.47747

ANOVA

	DF	Sum of Squares	Mean Square	F Value	Prob>F
A	1	280.89518	280.89518	12.68673	9.50083E-4
B	1	448.23595	448.23595	20.24473	5.51936E-5
C	1	474.6465	474.6465	21.43757	3.65475E-5
B*B	1	99.7486	99.7486	4.50518	0.03988
A*B	1	52.31582	52.31582	2.36286	0.13194
A*C	1	135.56664	135.56664	6.12291	0.01757
Error	41	907.77565	22.14087		
Total	47	2399.18434			

Fitted Plot

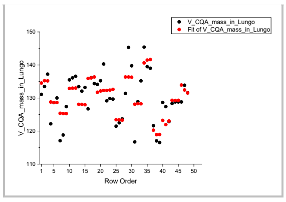

Residual Plot

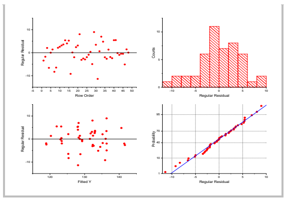

Effects Plot

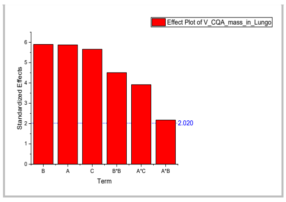

Main Effects Plot

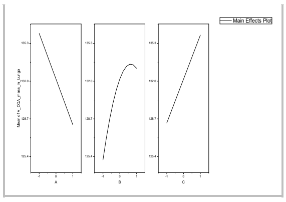

2-way Interaction Plot

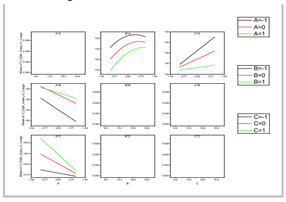

4.2 TDS - Ristretto

Coded Coefficients

	Value	Standard Error	95% LCL	95% UCL	t-Value	Prob > |t|
(Intercept)	2.96292	0.02332	2.91579	3.01005	237.76419	1.20275E-64
A	-0.16404	0.01864	-0.20171	-0.12638	-13.16369	4.0089E-16
B	0.0152	0.01602	-0.01718	0.04758	1.2197	0.22972
C	0.09076	0.01831	0.05376	0.12776	7.28306	7.59026E-9
A*A	0.0535	0.03354	-0.01429	0.12129	4.293	1.0891E-4
B*B	-0.08229	0.03022	-0.14336	-0.02122	-6.60325	6.71862E-8
C*C	0.03206	0.03148	-0.03157	0.09569	2.57275	0.01391
A*B	0.0562	0.01939	0.01702	0.09538	4.50949	5.56724E-5

Statistics

	TDS_mass_in_Ristretto
Degress of Freedom	40
Root Mean Square of Error	0.08634
R-Square	0.7835
Adj. R-Square	0.74561
Residual Sums of Squares	0.29816
Predicted. R-Square	0.68656

ANOVA

	DF	Sum of Squares	Mean Square	F Value	Prob>F
A	1	0.76563	0.76563	102.71433	1.31075E-12
B	1	0.0031	0.0031	0.41567	0.52279
C	1	0.17323	0.17323	23.23928	2.09272E-5
A*A	1	0.01593	0.01593	2.13669	0.15162
B*B	1	0.05286	0.05286	7.09186	0.0111
C*C	1	0.00563	0.00563	0.75591	0.3898
A*B	1	0.06264	0.06264	8.403	0.00605
Error	40	0.29816	0.00745		
Total	47	1.37717			

Fitted Plot

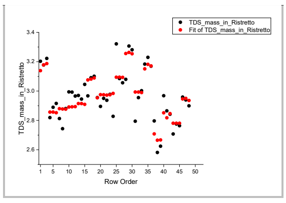

Residual Plot

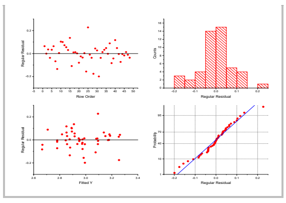

Effects Plot

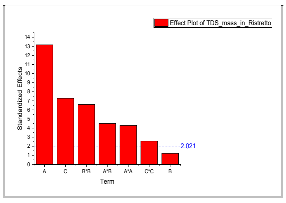

Main Effects Plot

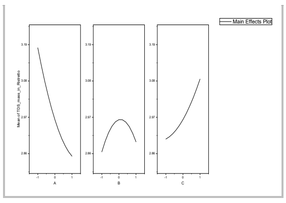

2-way Interaction Plot

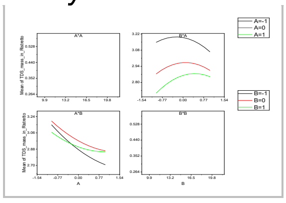

4.2 TDS - Espresso

Coded Coefficients

	Value	Standard Error	95% LCL	95% UCL	t-Value	Prob > |t|
(Intercept)	3.92539	0.02949	3.86584	3.98494	239.40909	3.95065E-66
A	-0.13786	0.02243	-0.18316	-0.09256	-8.40814	1.83557E-10
B	0.04416	0.02096	0.00183	0.0865	2.69361	0.01019
C	0.14428	0.02396	0.09588	0.19267	8.79936	5.44718E-11
B*B	-0.13301	0.0378	-0.20934	-0.05667	-8.1121	4.65387E-10
C*C	0.05332	0.03965	-0.02676	0.13339	3.25175	0.0023
A*B	0.06349	0.02512	0.01277	0.11421	3.87219	3.80091E-4

Statistics

	TDS_mass_in_Espresso
Degress of Freedom	41
Root Mean Square of Error	0.1136
R-Square	0.68971
Adj. R-Square	0.6443
Residual Sums of Squares	0.52907
Predicted. R-Square	0.56044

ANOVA

	DF	Sum of Squares	Mean Square	F Value	Prob>F
A	1	0.44808	0.44808	34.72372	6.14873E-7
B	1	0.03763	0.03763	2.91624	0.09525
C	1	0.45402	0.45402	35.1845	5.41213E-7
B*B	1	0.1306	0.1306	10.12062	0.00279
C*C	1	0.02322	0.02322	1.79906	0.18721
A*B	1	0.08246	0.08246	6.38988	0.01543
Error	41	0.52907	0.0129		
Total	47	1.70506			

Fitted Plot

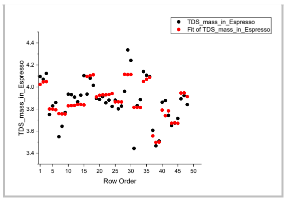

Residual Plot

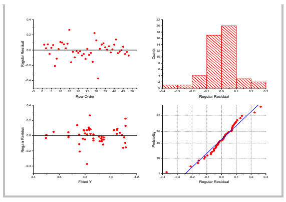

Effects Plot

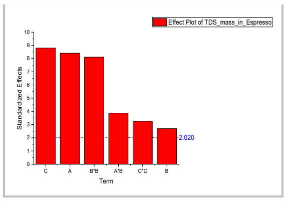

Main Effects Plot

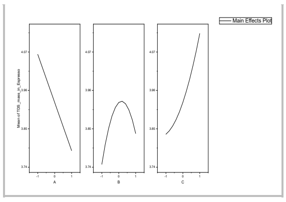

2-way Interaction Plot

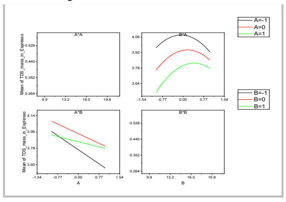

4.3 TDS - Espresso Lungo

Coded Coefficients

	Value	Standard Error	95% LCL	95% UCL	t-Value	Prob > |t|
(Intercept)	4.22727	0.0351	4.15633	4.29821	216.77954	4.84199E-63
A	-0.10842	0.02697	-0.16294	-0.0539	-5.55984	1.96247E-6
B	0.05376	0.02497	0.00329	0.10423	2.75682	0.00875
C	0.15812	0.02864	0.10023	0.216	8.10848	5.63771E-10
B*B	-0.16869	0.04499	-0.25963	-0.07775	-8.65065	1.06076E-10
C*C	0.06351	0.04733	-0.03215	0.15917	3.25678	0.0023
A*B	0.05925	0.0299	-0.00119	0.11969	3.03848	0.00418
A*C	-0.05714	0.03097	-0.11974	0.00545	-2.93045	0.00557

Statistics

	TDS_mass_in_Lungo
Degress of Freedom	40
Root Mean Square of Error	0.1351
R-Square	0.6357
Adj. R-Square	0.57195
Residual Sums of Squares	0.7301
Predicted. R-Square	0.45019

ANOVA

	DF	Sum of Squares	Mean Square	F Value	Prob>F
A	1	0.22816	0.22816	12.50035	0.00105
B	1	0.06771	0.06771	3.70978	0.06122
C	1	0.59515	0.59515	32.60632	1.20736E-6
B*B	1	0.21699	0.21699	11.88816	0.00134
C*C	1	0.02561	0.02561	1.40283	0.24324
A*B	1	0.07828	0.07828	4.28865	0.04486
A*C	1	0.06214	0.06214	3.4044	0.07244
Error	40	0.7301	0.01825		
Total	47	2.00414			

Fitted Plot

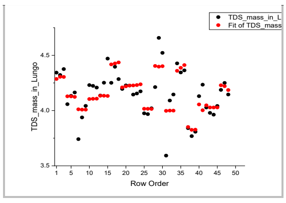

Residual Plot

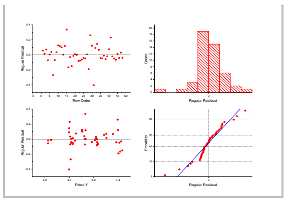

Effects Plot

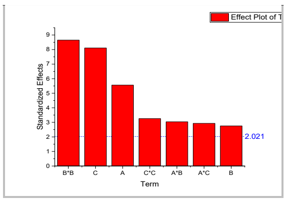

Main Effects Plot

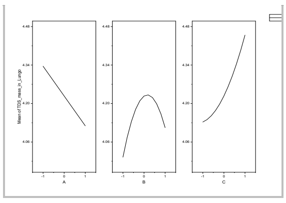

2-way Interaction Plot

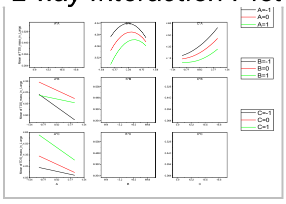

Supplement: Supplementary file 1 [file foods-12-02871-s001.zip › S4.2_Summary_OriginPro_Fitting_Evaluation_Calculations.pdf]
